# Hardware and Software Development for Isotonic Strain and Isometric Stress Measurements of Linear Ionic Actuators

**DOI:** 10.3390/polym11061054

**Published:** 2019-06-17

**Authors:** Madis Harjo, Tarmo Tamm, Gholamreza Anbarjafari, Rudolf Kiefer

**Affiliations:** 1 Institute of Technology, University of Tartu, Nooruse 1, 50411 Tartu, Estonia; madis.harjo@gmail.com (M.H.); tarmo.tamm@ut.ee (T.T.); 2 iCV Research Lab, Institute of Technology, University of Tartu, Tartu 50411, Estonia; shb@ut.ee; 3 Conducting polymers in composites and applications Research Group, Faculty of Applied Sciences, Ton Duc Thang University, Ho Chi Minh City 850000, Vietnam

**Keywords:** IIECMS, MWCNT-CDC fibers, PPy/DBS linear films, uncertainty measurements

## Abstract

An inseparable part of ionic actuator characterization is a set of adequate measurement devices. Due to significant limitations of available commercial systems, in-house setups are often employed. The main objective of this work was to develop a software solution for running isotonic and isometric experiments on a hardware setup consisting of a potentiostat, a linear displacement actuator, a force sensor, and a voltmeter for measuring the force signal. A set of functions, hardware drivers, and measurement automation algorithms were developed in the National Instruments LabVIEW 2015 system. The result is a software called isotonic (displacement) and isometric (force) electro-chemo-measurement software (IIECMS), that enables the user to control isotonic and isometric experiments over a single compact graphical user interface. The linear ionic actuators chosen as sample systems included different materials with different force and displacement characteristics, namely free-standing polypyrrole films doped with dodecylbenzene sulfonate (PPy/DBS) and multiwall carbon nanotube/carbide-derived carbon (MWCNT-CDC) fibers. The developed software was thoroughly tested with numerous test samples of linear ionic actuators, meaning over 200 h of experimenting time where over 90% of the time the software handled the experiment process autonomously. The uncertainty of isotonic measurements was estimated to be 0.6 µm (0.06%). With the integrated correction algorithms, samples with as low as 0 dB signal-to-noise ratio (SNR) can be adequately described.

## 1. Introduction

Ionic actuators are an interesting class of materials which can change their shape over electrochemical stimuli [1]. “Ionic” means that these materials rely on ions (from electrolyte) to carry out their shape change. These materials are also called “artificial muscles” [2] due to relative similarity to the behavior of natural muscles. Applications can be found in soft robotics [3,4], smart textiles [5], energy harvesters [6,7], and biomedical applications [8]. From the variety of materials belonging to this class, conducting polymers [9] and multiwall carbon nanotube/carbide-derived carbon (MWCNT-CDC) fibers [10] were chosen, which differ in their actuation mechanism and formation. Conducting polymers, such as polypyrrole, are a class of materials that follow redox-active processes (Faradaic actuators) [11]. During oxidation a positive charge is formed on the PPy network, in order to maintain the electroneutrality, counterions (anions with solvent) move into the PPy film [12]. Consequently, the polymer film swells (shape change), and during reduction those anions leave the film (anion-driven actuator). The whole process is reversible. Alternatively, if the anions are too large or too strongly interacting, like dodecylbenzene sulfonate (DBS), they become immobile inside the PPy film, while during reduction the charge of DBS^-^ anions is compensated by the ingress of cations into the film (cation-driven actuators, like PPy/DBS [13]). MWCNT-CDC fibers are formed by dielectrophoresis [14], where the fiber is drawn from a droplet of mixed MWCNT and CDC solution over a sharp needle under AC voltage. The MWCNT-CDC actuation mechanism follows the electrical double layer (EDL) processes (non-Faradaic actuators [15]), where the charging of the double layer results in C-C bond length change [16], but the actuation is mostly explained by ion migration [17,18].

For the development and characterization of actuator materials, an accurate measurement setup is an important tool. There have been several hardware constructions using different sensors, for example isometric transducers to measure force, and isotonic transducers to measure strain. For each sensor system, an individual experimental setup is required [19,20]. Recently, a unique hardware setup based on linear variable distance transducers [21] was reported for the measurement of the length change (strain) of conducting polymer devices. The setup consisted of a beam balance with constant weight on one side and the PPy helix tube on the other side. The disadvantage of this setup is the absence of any isometric (force) measurements. Some other constructions have been proposed based on optical signals (lasers) [22], whereas on the end of the hung film a thin wire suspends a reflecting plate through a pinhole of the cell and the up and down movement can be measured by laser displacement. In general, the combination of an isometric transducer with a linear actuation stage (LAS) has been shown to be capable of strain and stress measurements without changing the setup [23,24,25]. The main problem with all these hardware setups is the neglect of synchronization between the driving potentiostat and the linear actuation device. 

Our main interest, therefore, was to focus on a suitable software solution to perform synchronized isotonic and isometric measurements at the same time scale. Here, we want to demonstrate the suitability of the software solution on two different materials: one having high strain and stress—the conducting polymer (PPy/DBS), the other pushing the limitations of the software solution with low strain—the MWCNT-CDC fiber. Numerous manufacturers of stress and strain measurement equipment have advertised various software solutions, which provide isotonic or isometric measurement functionalities with driving signal generation possibilities [26,27,28]. The primary drawback of these systems, which in general are composed of two different devices (a potentiostat, a linear muscle analyzer with force sensor), is that the devices start and run independently from each other, [29] leading to disconnected time scales/time frames of the measurements— meaning poor synchronizability. Aurora Scientific [27] does provide a complete set of hardware (isotonic/isometric sensor, signal generator, data acquisition device) with a LabVIEW-based software (named dynamic measurement control (DMC)) that controls the hardware resources, executes experiments, and analyzes measured data [27,30,31]. Despite the apparent suitability of the DMC software and other similar products, these solutions are rarely available as open source. Even if they were, the isotonic/isometric measurement software solutions are directly designed for a specific hardware setup [28,32]. Another drawback is the amount of data, collected in binary format and in MB sizes, as the lowest sampling rates available are in the order of 1000 points/s, which make the data analysis rather complicated. Therefore, our interest was to obtain a flexible isotonic and isometric measurement solution that can be composed of devices from different manufacturers and handle data in a manageable text file format in kB scale. The most resource-efficient solution was to develop a custom software for running the desired experiments meeting the requirements. 

Here, we present the resulting measurement system and demonstrate its performance on two model ionic actuator systems—a conducting polymer film and a carbon composite fiber. 

## 2. Material and Methods

### 2.1. Materials

Solvents: propylene carbonate (PC, 99%) and ethylene glycol (EG, 99.8%) were purchased from Fluka (Bucharest, Romania). Pyrrole (Py, ≥ 98%, Sigma Aldrich, Taufkirchen, Germany) was vacuum-distilled prior to use and stored at a low temperature in the dark. Sodium dodecylbenzene-sulfonate (NaDBS, technical grade) and bis(trifluoromethane) sulfonamide lithium salt (LiTFSI, 99%), polyvinyl pyrrolidone (PVP, average mol. wt. 40,000) were acquired from Sigma Aldrich (Taufkirchen, Germany). MilliQ+ water was used for aqueous solutions. Amorphous titanium carbide (TiC)-derived carbon (CDC TiC-800) was purchased from Skeleton Technologies Ltd (Großröhrsdorf, Germany). Multiwall carbon nanotubes (MWCNT) were purchased from Sigma Aldrich(Taufkirchen, Germany) (O.D. × I.D. × L = 7–15 nm × 3–6 nm × 0.5–200 µm).

### 2.2. Formation of Ionic Electroactive Materials

#### 2.2.1. Multiwall Carbon Nanotube/Carbide-Derived Carbon (MWCNT-CDC) Fiber

The MWCNT and CDC material were dispersed in deionized water containing PVP as the surfactant and sonicated with a UPS200S (Hielscher, Teltow, Germany) ultrasonic processor for 30 min at 50% amplitude. PVP, CDC, MWCNT, and water were mixed at a ratio of 1:2 and: 4:1500 (wt %), respectively. The surfactant was added to stabilize the dispersion for extended periods. Fibers were prepared according to the method described recently [14]. Briefly, the tip of a chemically etched (diameter 0.1 µm) tungsten needle was inserted into a droplet of MWCNT-CDC suspended in deionized water deposited onto a stainless-steel plate. Then, an AC voltage was applied between the tungsten tip and the metal plate (0–350 Vpp, 2 MHz), and the tungsten tip was withdrawn until a MWCNT-CDC fiber of the desired length was formed. The initial spacing between the needle and the plate electrode (i.e., the height of the droplet) was 3 mm. The retraction of the needle was performed with an M-413.3PD precision stage (Physik Instrumente, Karlsruhe, Germany). MWCNT-CDC fibers with embedded CDC particles in the range of 25% were obtained (with an average diameter of 150 µm) and dried in an oven at 100 °C.

#### 2.2.2. Polypyrrole Doped with Dodecylbenzene-Sulfonate (PPy/DBS)

PPy/DBS was polymerized galvanostatically at 0.1 mA cm^−2^ (40,000s) in a 2-electrode cell with a stainless-steel mesh counter electrode and a stainless-steel sheet working electrode (18 cm^2^) in a 0.1 M NaDBS, 0.1 M Py, in EG/Milli-Q (1:1) mixture. The temperature of the polymerization was −40 °C. The obtained PPy/DBS films were washed in ethanol to remove excess of pyrrole with additional washing steps in MilliQ+ water to remove excess of NaDBS, and dried in an oven. The film, in thickness of 18.5 µm, were then stored in LiTFSI propylene carbonate (0.2 M) electrolyte.

### 2.3. Linear Muscle Analyzer Set up

**Figure 1:**
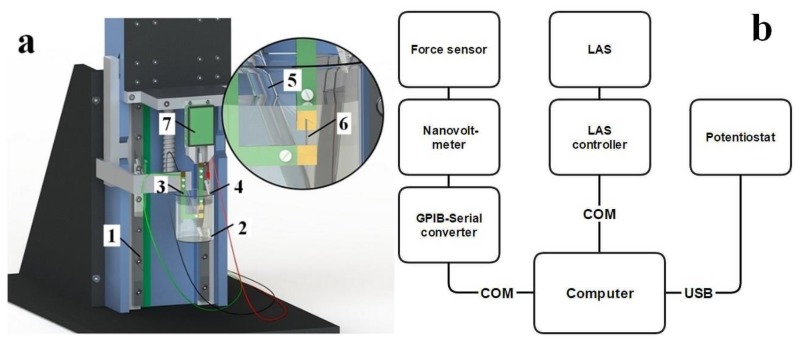
**a**: Stress and strain measurement (linear muscle analyzer) setup [10]. (1) Linear Actuation Staging (LAS), (2) beaker with electrolyte, (3)(4)(5) electrodes of the potentiostat, (6) ionic electroactive material sample, (7) force sensor. **b**: Connection hierarchy of the devices used for measurement setup.

To measure strain and stress without changing the test setup, a Linear Actuation Staging (LAS) with an isometric transducer (force sensor) were combined. The setup is shown in Figure 1a and the connection hierarchy in Figure 1b.

Figure 1a shows that the sample (6) is attached between two clamps. The lower clamp is static and is fixed to the chassis (1) of the LAS (Linear actuation stage). The upper clamp is attached to a force sensor (7) which is mounted on a plate of the LAS (Physik instrumente M-414.3PD, min step size 0.5 µm, Karlsruhe, Germany) that enables it to perform linear movements. Figure 1b shows the hierarchy of the devices. The signal of the force sensor (Panlab TRI202PAD, Barcelona, Spain) is read by a voltmeter (Keithley 2182A, Beaverton, United States) which exchanges data with the computer over a General-Purpose Interface Bus (GPIB) to Universal Serial Bus (USB) converter (Prologix Rev 6.4.1, Asheville, North Carolina). The LAS (Physik Instrumente M-414.3PD, Karlsruhe, Germany) is controlled by a controller (Physik Instrumente C-863, Karlsruhe, Germany), which accepts commands from the computer via a serial port. Lastly, the potentiostat (Biologic PG581, Göttingen, Germany) is connected to the computer using a USB interface.

To examine the samples, PPy/DBS films and MWCNT-CDC fibers were cut in strips of 1.0 cm * 0.1 cm and fixed between the force sensor and on the fixed arm with gold contacts that served as a working electrode in the linear muscle analyzer setup (Figure 1). The initial length of the films between the clamps was 1 mm. The strain, ε, was calculated from the formula *ε = Δl/l,* where *Δl* refers to *l –l_1_* with *l* as the original length of the film (1 mm) and *l_1_* the change of length obtained from isotonic measurements. The load applied on PPy/DBS films was 6 g (58.8 mN, 3.2 MPa) and the load on MWCNT-CDC films was 60 mg (0.6 mN, 33.2 kPa). For isometric (force) measurements, the stress, *σ,* was calculated using the formula *σ = F/A,* where *F* is the force in N acting on an object (F = m*g with *m* the mass and *g* means acceleration due to gravity as a constant 9.8 m s^−2^) and *A* represents the cross-sectional area of the object (width * thickness). A platinum sheet was used as the counter electrode in the measurements cell and Ag/AgCl (3M KCl) as the reference electrode. For PPy/DBS films and for MWCNT-CDC fibers, 0.2 M LiTFSI propylene carbonate solution was applied. For isotonic and isometric measurements, cyclic voltammetry (scan rate 5 mV s^−1^) was used for driving in the potential range of 0.65 to −0.6 V.

#### Characterization of the Materials

PPy/DBS films and MWCNT-CDC fibers were examined by scanning electron microscopy (Helios NanoLab 600, FEI, Hillsboro, OR, USA). The electrical conductivity of the fibers was measured by a simple two-point probe method.

### 2.4. Linear Muscle Analyzer Software Description

The software of the measurement system was developed [33] in the LabVIEW 2015 environment, and it provides different control modes (Figure S1), which allows it to calibrate the force sensor, test sample elasticity, run electrochemical analysis with the potentiostat (real time measurements), and measure weight change (stress) and length change (strain). For real time measurement requirements, the shortest data acquisition period must be 1s, and after the user has activated an experiment, the devices must be initialized in 10s. 

#### 2.4.1. Calibration of the Force Sensor

Calibration of the force sensor is necessary for converting the force sensor’s output voltage into values, which represent milligrams. To operate the software, the measurement called “bias” will calibrate the force sensor by measuring the baseline voltage arising from no load on the force sensor. In the next step, the signal arising from different known weights is measured. Equation (1) gives the voltage-to-milligram transition coefficient C:

(1) $$C=\;\frac{m_{w}}{U_{w}-U_{B}}$$

where, *m_w_* is the weight of the calibration load in milligrams, *U_w_* is the voltage of the force sensor’s output which is measured by voltmeter when the calibration load is applied, and *U_B_* is the bias voltage. The coefficient is stored in a .txt file. In order to calculate the force from raw voltage measurements, Equation (2) is presented:

(2) $$F=C\left(U-U_{B}\right)$$

where, *F* is the calculated force, and *U* is the potential measured from the force sensor’s output.

#### 2.4.2. Elasticity Measurements

Elasticity determination is essential to obtain linear length change (strain) results. In all available commercial muscle analyzers, only the calibration of the force sensor is performed by a defined weight, which will provide uncertainties in real measurements due to the fact that the elastic modulus of an ionic actuator changes during charging/discharging cycles [34,35]. The elasticity estimation of an artificial muscle is based on Hooke’s law (Equation (3)):

(3) F= −kX

where, *F* is the force needed to extend or compress an object by some distance *X* and k is a constant factor characterizing object elasticity. If a derivative of the force is taken with respect to distance, Equation (4) is obtained:

(4) $$k=-\;\frac{\partial F}{\partial X}$$

The idea was to perform a known movement with LAS and measure the changes of force. Figure S2 shows the block diagram of the software with the respective steps in the description of the algorithm.

#### 2.4.3. Initialization and Experiments with the Potentiostat

The potentiostat controls the electrochemical processes of ionic actuators, which translate the electrical signal (potential, current) to shape change, which is measured as force (weight change) or strain (length change). 

The initialization process of the potentiostat is based on three steps: (a) reading the variables describing how the experiment will be performed, (b) setting up the potentiostat for the experiment, and (c) starting the experiment. The setup variables are directly read from the user interface from user-controllable variables. The experiment type is selected by the user from a set of three: cyclic voltammetry (CV), chronoamperometry (CA), and chronopotentiometry (CP). The block diagram for the potentiostat is shown in Figure S3a, strain and stress measurements have the same basic structure.

#### 2.4.4. Strain and Stress Measurements

Strain experiments also include an additional muscle length controlling logic, as the upper clamp is moved by the LAS plate to maintain constant force during the scan. The magnitude of the required movement by the LAS constitutes the isotonic strain. In order to keep a constant force, a proportional controller (P controller) is implemented, as presented in Equation (5), which derives from Equation (4):

(5) $$\Delta X=-k^{\prime }\Delta F$$

where, the gain k´= (k + u_k_), and u_k_ is the uncertainty of k. The addition of u_k_ to k assures that no faulty steps are done in a case when $k≅\;u_{k}.$ The input (ΔF) is the difference between the measured force and the set state (force value which the P controller is intended to track). The output is a step size (ΔX) needed to maintain the muscle in the set state of force. The gain (k) for the P controller is the elasticity coefficient of a muscle, whose strain is to be measured. The minimum steps size that the LAS can carry out is Δl_min_ = 0.5 µm. In case the ΔF is smaller then:

(6) $$\Delta F_{min}=\frac{\Delta l_{min}}{-k^{\prime }}$$

No movement is carried out since it would require a step size < Δl_min_. In such cases, the electroactive material has linear displacement below the resolution of the LAS, the actual force can differ from the set value and is equal to ΔF_min_. The detailed description of the block diagram for strain measurement is given in Figure S3b.

**Figure 2:**
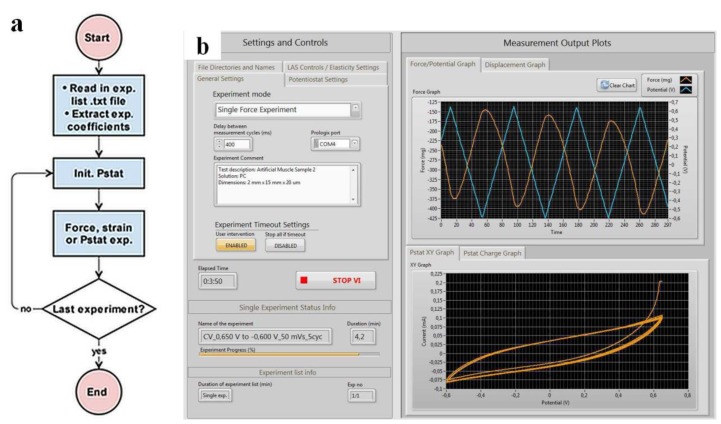
**a:** Block diagram of the automated multiple stress or strain measurement processes, and **b**: Layout of the graphical user interface (single force experiment) of the developed software.

The automated multiple stress or strain measurement process is shown in Figure 2a and the user interface of the isotonic (displacement) and isometric (force) electro-chemo-measurement software (IIECMS) [33] is presented in Figure 2b. The IIECMS program is designed for measurement automation with the additional function of avoiding user errors in mislabeling or accidentally selecting wrong settings for the experiments.

The block diagram of the automated measurement process (Figure 2a) reads the experiment list and coefficients from a previously generated .txt file. Coefficients, which describe the experiments, are extracted and placed into a temporary array. The program enters into a for-loop, where the potentiostat is initialized and the user-selected measurement is executed. The number of cycles in the for-loop is equal to the amount of experiments on the list.

The graphical user interface (GUI) in Figure 2b shows three main sections. Section 1 includes all controls, such as general settings, file direction and names, potentiostat settings, and motor controls/elasticity measurement. Section 2 includes a set of indicators of experiment progress, such as ongoing experiment and experiment list info’s. In Section 3, four graphs are included that plot measurement data.

## 3. Results on Model Systems and Discussion

The IIECMS program was adapted on real sample measurements. Two different types of test samples were measured (conducting polymers PPy/DBS and MWCNT-CDC fibers) to show isotonic and isometric measurement results. In the case of the conducting polymer, the isotonic length change or displacement amplitude was more than 20 times higher (referred to as high signal-to-noise ratio ca. 20 dB) than the actuation resolution of the LAS (Δl_min_ = 0.5 µm). The second measurement represents a situation where the displacement amplitude is comparable to Δl_min_ (referred to as low signal-to-noise ratio) and a measurement resolution refining technique is described.

Over 50 different isometric and istotonic measurements were made on ionic material samples, meaning about 200 h of measurement time. The minimum data acquisition period of this measurement setup was 160 ms.

### 3.1. Characterization of Ionic Actuator Materials

**Figure 3:**
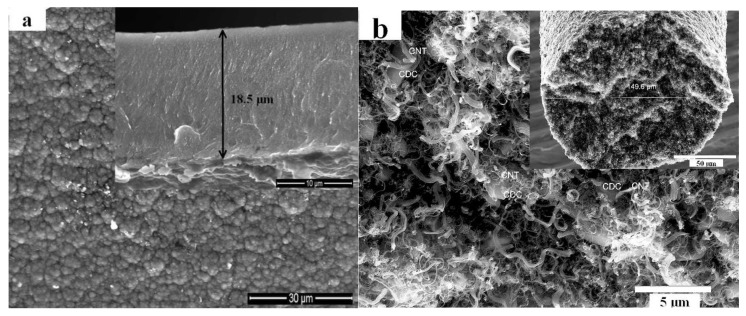
Scanning electronic microscopy (SEM) images of **a**: Polypyrrole Doped with Dodecylbenzene-Sulfonate (PPy/DBS) surface (scale bar 30 µm) with inset—the cross-section (scale bar 10 µm), and **b**: MWCNT-CDC fiber surface (scale bar 5 µm) with inset—cross-section (scale bar 50 µm).

In the case of PPy/DBS, the DBS^-^ counterions are immobilized during electropolymerization [36] and upon discharging, their negative charge is compensated by (solvated) cations, as shown before [37,38]. In the case of MWCNT-CDC fibers, the actuation mechanism is based on the charging of the electric double layer (non-Faradaic actuator) [10]. Figure 3 shows the scanning electron microscopy (SEM) images of the two different electroactive materials applied in this work.

The surface of the PPy/DBS film (Figure 3a) showed a typical cauliflower structure [39], with the cross-section showing a thickness of 18.5 µm. The diameter of the MWCNT-CDC fiber, as measured from the cross section (inset in Figure 3b), was 149.6 µm, the solid particles partly seen represent CDC surrounded by MWCNT material [10] (marked in Figure 3b). The conductivity of the PPy/DBS films was 0.5 ± 0.04 S cm^−1^ while MWCNT-CDC fiber had 13.5 ± 7 S cm^−1^, in line with those shown before [10]. When it comes to linear actuation measurements, the mechanical properties, such as brittleness and elasticity, are important [13]. PPy/DBS films were easy to handle for fixing them on the force sensor and the upper clamp (Figure 2a), while the MWCNT-CDC fiber was very brittle and required extremely delicate handling. 

### 3.2. High Signal-to-Noise Ratio Isotonic and Isometric Measurements

**Figure 4:**
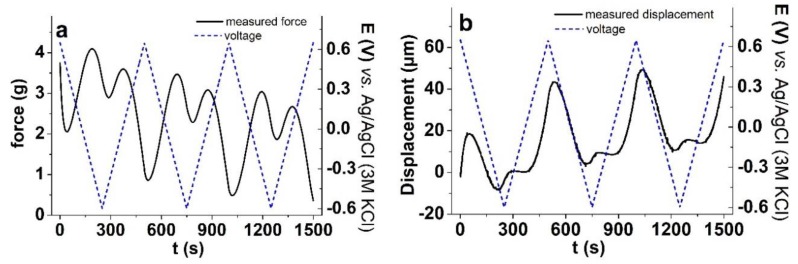
Cyclic voltammetry measurements (scan rate 5 mV s^−1^, 3 cycles) of PPy/DBS films in LiTFSI-PC electrolyte in voltage potential (dashed) range 0.65 to -0.6V showing in **a**: isometric (measured force, black line), and **b**: isotonic (measured displacement, black line) results against time.

The performance of the setup was demonstrated on PPy/DBS samples in LiTFSI-PC solution in the potential range of 0.65 to −0.6 V under isometric (Figure 4a) and isotonic (Figure 4b) modes. This relates to case one, where isotonic length change or displacement amplitude is about 2 orders of magnitude larger than the minimal step size of the LAS (Δl_min_ = 0.5μm). 

Figure 4a shows the force (weight change) results of the isometric measurements of PPy/DBS films driven by cyclic voltammetry. The maximum stress for this film was found in range of 0.9 MPa. In the case of PPy/DBS, which in general is a typical cation-driven actuator due to the immobile DBS^-^ ions left in the PPy network, the actuation in LiTFSI-PC is a special phenomenon where the DBS^-^Li^+^ ion pairs partly become undissociated in propylene carbonate solvent due to its aprotic nature [13,40]. Therefore, new places of the PPy/DBS film are oxidized and the solvated counter ions TFSI^-^ can enter the film and swell the film at oxidation (expansion, seen in Figure 4b). Our setup was easily able to detect and distinguish between the two processes taking place during one charging cycle. During the first scan (Figure 4a), the potential went from 0.65 to −0.6 V and the force decreased, then increased and finally showed a small decrease at −0.65 V. The contraction corresponds to a small cation involvement during reduction (seen in Figure 4b of displacement measurements, maximum strain in range of 0.6%). During the next cycles, the force was reduced, corresponding to increased displacement (strain). In both displacement and force graphs, creep [24] can be detected, which means that the “neutral” position of the linear PPy/DBS actuator changes, a relatively common behavior for ionic electroactive materials [41]. The phase shift (Figure 4b) of displacement in respect to the driving signal voltage is due to the participation of cations, which in the initial phase of a cycle lead to volume contraction, before the anions take over [13]. 

Upon very careful observation, one can see some noise appearing in Figure 4b, for example at position 200 s, 700 s, and 1200 s. The noise is introduced when the actual elasticity coefficient, which is used for P controller, temporarily differs from the measured value. This is due to the ion–matrix interactions. In extreme cases, when the elasticity coefficient is measured to be much lower, the P controller (Equation (6)) starts to generate. The elastic coefficient, k, was calculated from Equation (3), giving for PPy/DBS, before charging/discharging, the value of 239 mg/µm and after charging/discharging (50 actuation cycles), the value of 134 mg/µm. As it has been shown previously, the elastic modulus of conducting polymers changes during actuation cycles [42] was also effected by the nature of the solvent applied [43]. Therefore, as seen from the above results, the determination of the elastic coefficient to operate isotonic measurements is needed in order to measure meaningful data.

### 3.3. Low signal-to-Noise Ratio (SNR) Isotonic Measurements

**Figure 5:**
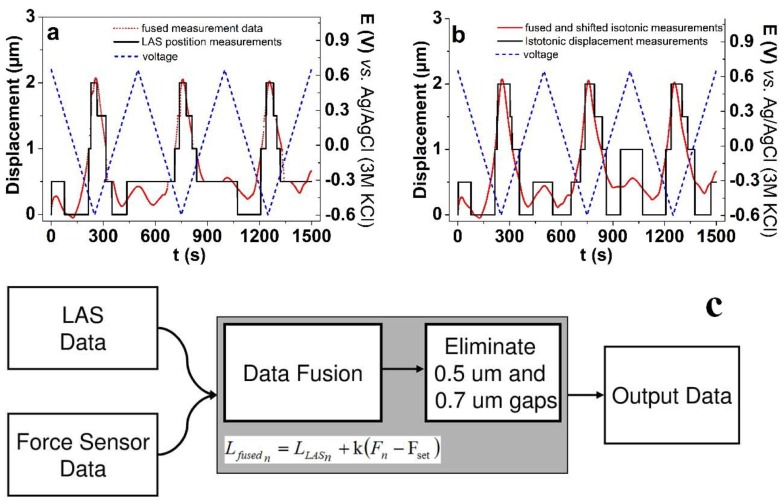
MWCNT-CDC fiber under cyclic voltammetry (scan rate 5 mV s^−1^ (3 cycles), potential range 0.65 to −0.6 V) in LiTFSI-PC electrolyte. **a**: Near 0 dB SNR isotonic displacement measurement results (black line) and force sensor data fused with measurements data (red points) with voltage (dashed, blue). **b**: Fused and shifted isotonic measurements (red points) after being processed by slack correction algorithm with voltage (dashed, blue) compared to isotonic displacement (original LAS position measurements). **c**: Near 0 dB SNR strain measurement data correction algorithm. LAS and force sensor data (inputs) are fused via Equation (7). Then 0.5 um and 0.7 um gaps are eliminated, and resulted data is outputted.

To examine materials which have very low displacement amplitude, comparable to Δl_min_ (near 0 dB, SNR) MWCNT-CDC fibers were applied in isotonic displacement measurements conducted with cyclic voltammetry (scan rate 5 mV s^−1^) but with the same electrolyte and potential range seen in Figure 4b. The results are presented in Figure 5. 

Figure 5 shows the case where the displacement (isotonic measurements) of the ionic electroactive material is in the limitation of the measurement setup. Figure 5a shows the raw measurements of MWCNT-CDC actuators under the cyclic voltammetric technique, which shows a rough estimate about the specimen’s isotonic actuation properties. The graph in Figure 5a in the present form cannot be used in further data analysis. To give a better interpretation of the results, the force sensor data can be fused with isotonic displacement measurements. We know from the elastic coefficient measurements that the change in force is proportional to the change of length, therefore the fusion can be implemented as Equation (7):

(7) $$L_{fused_{n}}=\;L_{LAS_{n}}+k\left(F_{n}-F_{set}\right)$$

where, *n* is the index of the measurement data sample, $L_{LAS_{n}}$ is the isotonic displacement measured by LAS’s controller, *F_n_* is the measured force, and *F_set_* is a set force which is constant throughout the experiment. Taking a closer look at the fused measurements shown in Figure 5a, there is some sort of ambiguous slack in the LAS actuation system, for example in the range of 310 s to 320 s, in the form of a 0.5 µm gap (comparable with Δl_min_) between the measurement points, whereas similar gaps appear at other time steps as well. We assume that the slack might be a combination of the slack in the muscle samples clamping system, the internal properties of the samples, and the LAS controller position measurement or actuation errors. We assume that since the combined slack is comparable with Δl_min,_ it is most likely caused by the LAS controller error. Figure 5b shows the fused isotonic displacement (red points) after being processed by a slack correction algorithm in comparison to the original LAS position measurements. Figure 5c gives the data correction algorithm in the near 0 dB SNR displacement measurements by using two cycles, where the first removed the 0.5 µm gaps and the second the 0.7 µm gaps. After applying this algorithm, the results of the displacement measurements of MWCNT-CDC fibers can be interpreted.

Figure 5a,b results in expansion at discharging (−0.65 V) in the propylene carbonate solvent, which was explained [10] by the anions being nearly immobile, whereas at discharging the (solvated) cations (Li^+^) balance the negative charge bringing along the length change of the actuator [44]. In the case of the MWCNT-CDC fiber studied in this research, the main expansion also appeared at discharging with displacement in the range of 2 µm (equivalent to 0.2% strain). It was also found that small expansion at oxidation in the range of 0.3 µm appeared, which we assume relies on a small expansion accompanying the charging process of the EDL formed [45].

### 3.4. Uncertainty Evaluation

**Figure 6:**
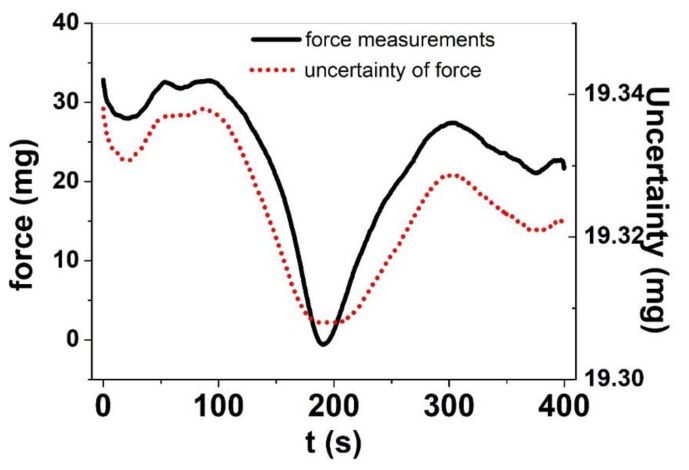
Cyclovoltammetric (scan rate 5 mV s^−1^, 3^rd^ cycle) isometric measurement results of MWCNT-CDC fibers in LiTFSI-PC electrolyte at potential range 0.65 to -0.6V, showing the force measurements (mg) (black line) and the uncertainty (B type) of the force measurements (mg) (red dotted).

The uncertainty of force measurements depends on the components [46] which are used in the force calculations. Since force is calculated based on Equation (2), the combined uncertainty is shown in Equation (8):

(8) $$\mu =\;\sqrt{{\left(\frac{\partial F}{\partial C}u_{c}\right)}^{2}+{\left(\frac{\partial F}{\partial U_{B}}u_{U_{B}}\right)}^{2}+{\left(\frac{\partial F}{\partial U}u_{U}\right)}^{2}+u_{Fd}^{2}}$$

where, *u_C_* is the uncertainty of the voltage-to-milligram coefficient, *u_UB_* is the uncertainty of the bias voltage, *u_U_* is the uncertainty of the potential measured from the force sensor, and *u_Fd_* is the uncertainty caused by the drift of force measurements. The expanded uncertainty of stress and strain measurements is stated as the standard uncertainty of measurement multiplied by the coverage factor *k* = 2, which for a normal distribution corresponds to a coverage probability of approximately 95%. The uncertainty of the force measurements is proportional to the voltage values measured from the force sensor, which can be seen from Equation (2). This means that small force amplitude signals have higher accuracy, whereas with the growth of signal amplitude, the measurement uncertainty increases. Figure 6 shows the case of small amplitudes in force (mg) for MWCNT-CDC fibers.

The MWCNT-CDC fiber (Figure 6) showed change in force in the range of 30.5 mg, which is translated to stress in the range of 17 kPa. There is a small decrease in force from the starting point of 33 mg to the end point (22.8 mg) of 10.2 mg. The decrease in maximum force belongs to the creep effect, which can appear if mixed ion involvement appears, which is also seen in recent research [47].

The discharging, therefore, leads to the decrease in force which is translated into displacement knowing the elastic coefficient of the MWCNT-CDC fiber (*k* = 13.5 mg/µm). According to the uncertainty measurement in Figure 6 based on Equation (8), the coefficient u_c_ was estimated to be 16.8 g/V and *u*_UB_ = *u*_U_ = 0.005 mV. The most important contributor to displacement measurement error is the resolution of the LAS position estimation system, which is 0.5 μm, therefore, the B type uncertainty of strain measurements without the assistance of data fusing (Equation (8)) is $k\frac{0.5\;µm}{\sqrt{3}}≅0.6$ μm. The data fusion contributes marginally (usually <0.05 μm) to the uncertainty of strain measurements, but it clarifies the interpretation of the results.

## 4. Conclusions

A custom software solution was designed and implemented for driving and measuring ionic elelctroactive material-based systems. The hardware setup combined separately acquired commercial off-the-shelf devices like a force sensor, a voltmeter, a linear actuation stage, and a potentiostat. To operate the electro-chemo measurement system in a synchronized manner, a new software with graphical interface was developed in LabVIEW. The IIECMS was made by combining software development kits of the implemented devices, creating algorithms for the execution of isotonic and isometric measurements, and automating the measurement and user input processes in LabVIEW. The maximum user-selectable measurement frequency for IIECMS was 160 ms. In order to operate the potentiostat in the LabVIEW, a respective set of software drivers were created with ActiveX-based SDK of the potentiostat. The IIECMS program was applied on PPy/DBS linear actuators revealing the first case of 20 dB high signal-to-noise ratio isotonic and isometric measurements with maximum force of 3 g (stress of 0.9 MPa) and strain in the range of 4.3% at oxidation. The system was easily capable of detecting mixed-mode driving and distinguishing between the cation and the anion flux induced actuation. The MWCNT-CDC fibers belonged to the second case of 0 dB low signal-to-noise ratio isotonic measurements. Partial discontinuity of the data indicated that the IIECMS setup has an occasionally appearing slack of approximately 0.7 µm, which can be eliminated with post experiment data processing. The displacement of the MWCNT-CDC fibers was found in the limitation of Δl_min_ (0.5 µm) with maximum displacement at a discharging of 2 µm (0.2 % strain). The accuracy of isotonic measurement was estimated to be in the range of 0.6 µm, with the largest contributor relying on the measurement error of the position estimation resolution of LAS. Fusing the output of different channels allows for successful operation even at such challenging conditions.

Future perspectives for the IIECMS program will be to fully automate the isotonic and isometric experiments. This means that the user has an option to run both experiment types in an automated sequence, thus eliminating the need for a user to measure sample elasticity. In principle, any type of voltage- or current-driven linear actuators in the form of strips, fibers, etc., could be studied with the IIECMS program.

## Supplementary Materials

The following are available online at https://www.mdpi.com/2073-4360/11/6/1054/s1. Figure S1 General structure of the developed software, Figure S2 Block diagram of the elasticity measurement process, Figure S3 Block diagram of potentiostat and strain measurement.
