# Supplementary material for: Hardware and Software Development for Isotonic Strain and Isometric Stress Measurements of Linear Ionic Actuators
Source: Polymers (Basel). 2019 Jun 17;11(6):1054. doi: 10.3390/polym11061054 (PMC6631421; doi:10.3390/polym11061054)
Supplement: Supplementary file 1 [file polymers-11-01054-s001.pdf]

Supplementary

Hardware and Software Development for Isotonic Strain and Isometric Stress Measurements of Linear Ionic Actuators

Madis Harjo[1], Tarmo Tamm[1], Gholamreza Anbarjafari[2], Rudolf Kiefer[3,*]

[1]Institute of Technology, University of Tartu, Nooruse 1, 50411 Tartu, Estonia

[2]iCV Research Lab, Institute of Technology, University of Tartu, Tartu 50411, Estonia

[3]Conducting polymers in composites and applications Research Group, Faculty of Applied Sciences, Ton Duc Thang University, Ho Chi Minh City, Vietnam

[*]Corresponding author, E-mail address: rudolf.kiefer@tdtu.edu.vn (R. Kiefer).

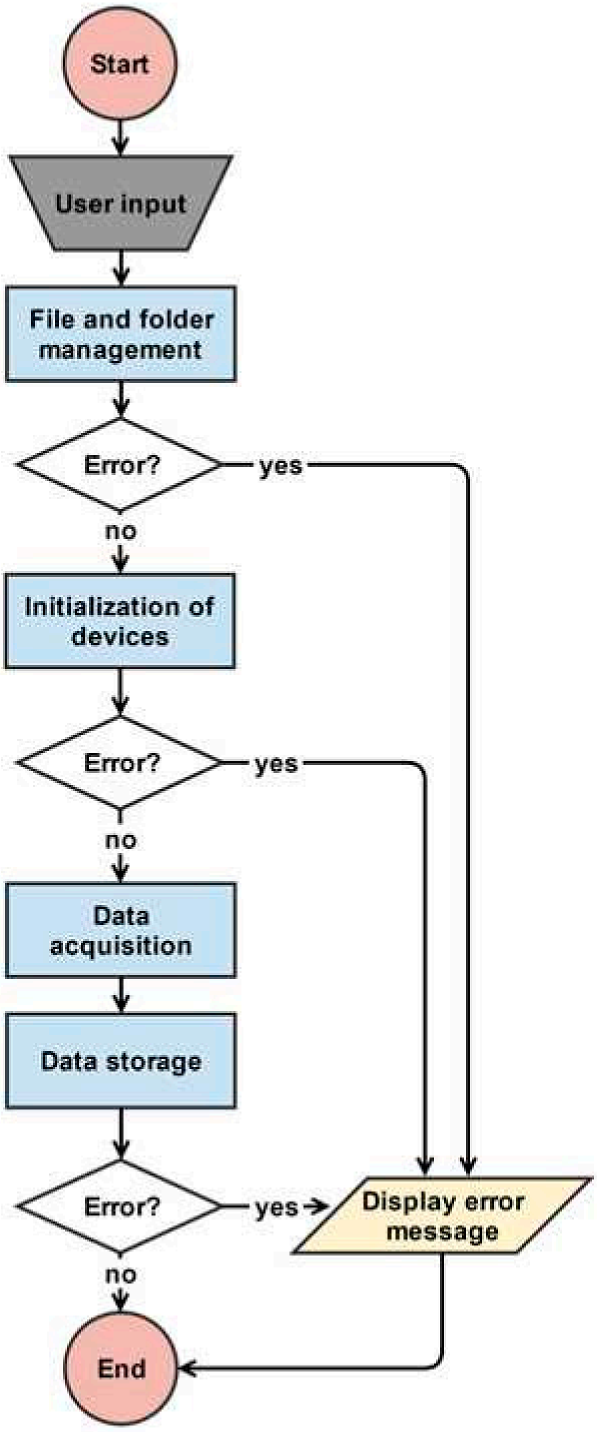

Figure S1: General structure of the developed software.

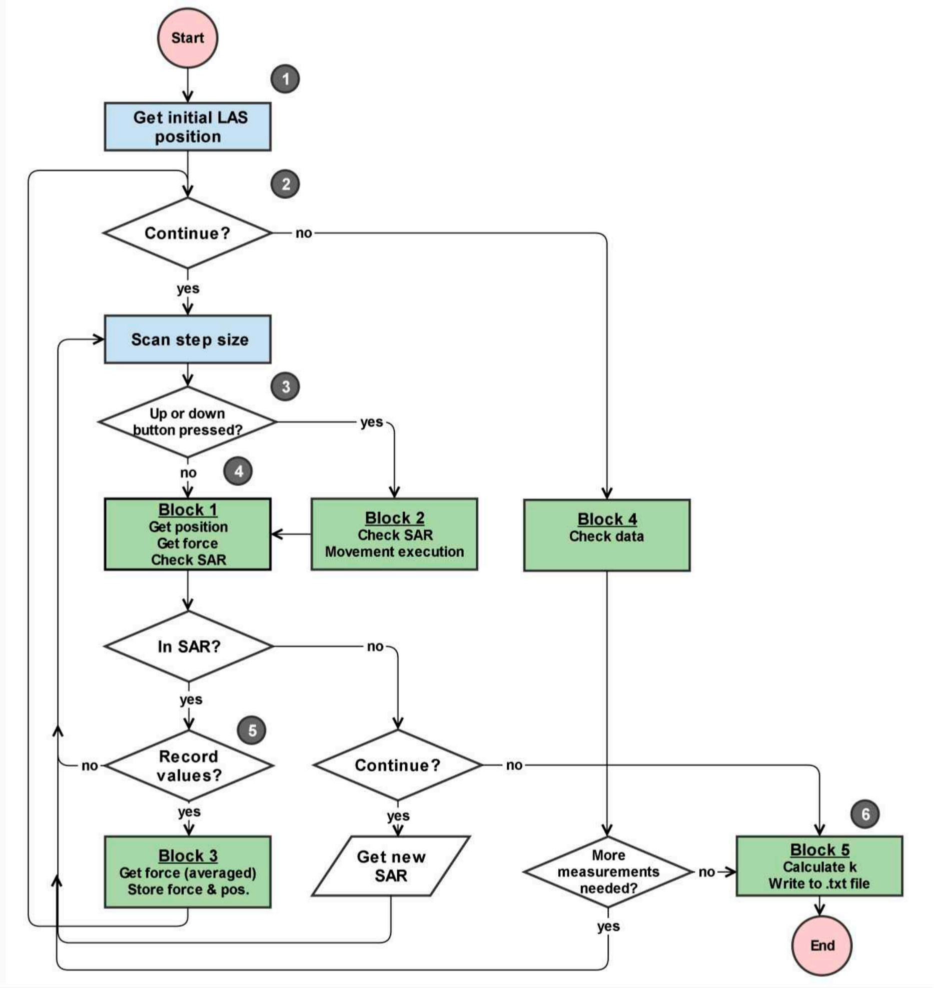

Figure S2. Block diagram of the elasticity measurement process. The numbers from (1) to (6) refer to according step in the description of the algorithm.

The step by step description of the algorithm is described in Figure S2. Step1 gives the obtained initial position of the linear actuation state (LAS), the value is stored as a constant. At step 2, the user dialog is open and the user is asked if new measurement cycles will be continued. In case that the user say no, the available measurement data is checked (block 4) if there enough data given to process. In case it is a dialog open with the information that the measurement should be stopped. If the user wants to continue, the processes in step 3 are carried out. In case there is enough data forto determine the elasticity coefficient, the process in step 6 is carried out. At step 3, the LAS step size is read in (can be changed by user even in runtime) and the buttons moving up and down are checked. If the LAS is moving up or down the program executes block 2, which is checking if the desired movement of LAS doesn’t exceed the safe actuation region (SAR) and in a positive case the movement will proceed. In case the SAR is exceeded, the movement will not be made and a warning is given to block 1 (shown in step 4).

In case up or down buttons are not pressed, the processes in step 4 are carried out. At step 4, the LAS position is acquired, the force is measured and the SAR conditions are checked (block 1). The processes in step 3 are performed. Step 6 describes the execution of block 5. The average force values with uncertainties and positions of LAS are taken from the temporary array. The elasticity and its uncertainty is calculated out of 2 measurement pairs (meaning 10 measurements were taken, 5 values of elasticity can be calculated). The elasticity and uncertainty calculation results are stored in a ".txt" file. The measurement process is finished.

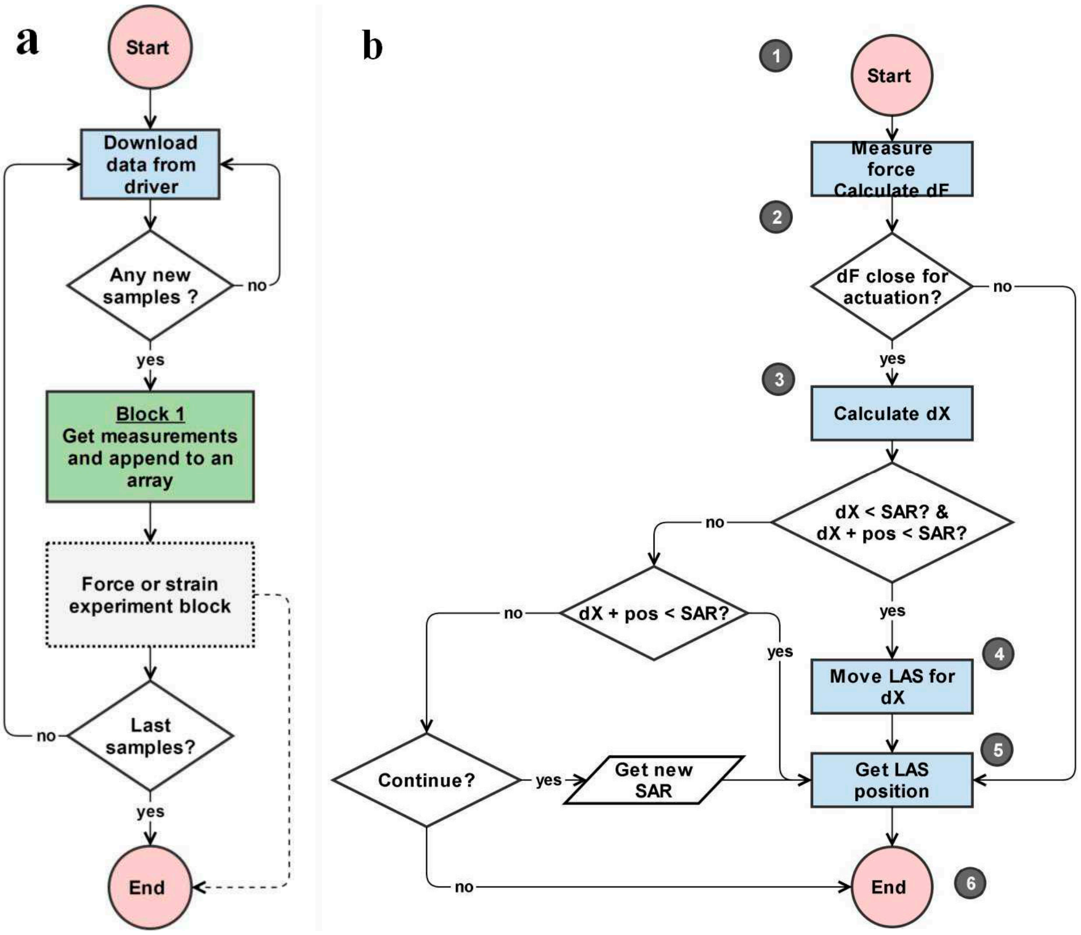

Figure S3: a: Block diagram of potentiostat experiment. Stress and strain experiments have the same basic structure if according commands are added. b: Block diagram of the strain measurement process. The numbers from (1) to (6) refer to according step in the description of the algorithm.

After initialization and setup of the experiment (Figure S3a), the main loop of the experiment program is entered. Firstly, a data downloading command is executed, which downloads any available data from potentiostat and retrieves the number of available samples. If there are any

new samples, the data (voltage, current, time) is appended to according storage arrays (block 1 in Figure S3a). The dashed arrow between force and strain measurement block and the end node in Figure S3a represents the case when strain experiment has exceeded the safe actuation boundary. At Figure S3b the block diagram of strain is presented in which in first stage the code started, then the force measured and ΔF is calculated. There two cases if $\Delta F > \frac{\Delta l_{min}}{-k'}$ then the process in step 3 (LAS movement is calculated) is carried out (Figure S3b) and if $\Delta F < \frac{\Delta l_{min}}{-k'}$ step 5 is addressed. To calculate the step size ΔX applying equation 6 an additional term of "Safe Actuation Region" (SAR) is included to avoid in the step size damaging of any equipment and measurement specimen due to unwanted commands. Under the condition that ΔX < SAR and ΔX + LAS position < SAR means that the step size not bigger than SAR and the movement process ΔX would not cause the LAS exist SAR then the process at 4 (Figure S3b) is carried out. If the condition described in 2 is not true then (ΔX + LAS position < SAR) = TRUE? is checked again and if true then step 5 (Position of the LAS is acquired from LAS's controller and stored) is carried out. In case it's not true the step 6 is carried out and program stopped or user is asked if experiments should be continued or not. In case the user want to continue a new value for SAR is asked and step 5 is carried out.
